# Comparative Genomic Analysis of Classical and Variant Virulent Parental/Attenuated Strains of Porcine Epidemic Diarrhea Virus

**DOI:** 10.3390/v7102891

**Published:** 2015-10-23

**Authors:** Fangzhou Chen, Yinxing Zhu, Meizhou Wu, Xugang Ku, Shiyi Ye, Zhonghua Li, Xiaozhen Guo, Qigai He

**Affiliations:** State Key Laboratory of Agricultural Microbiology, College of Veterinary Medicine, Huazhong Agricultural University, Wuhan 430070, China; chenfangzhou@webmail.hzau.edu.cn (F.C.); yingzizhu10@163.com (Y.Z.); wumeizhou@mail.hzau.edu.cn (M.W.); kuxugang84@163.com (X.K.); yeshiyi@webmail.hzau.edu.cn (S.Y.); yuxincangqiong@163.com (Z.L.); gxz0821@163.com (X.G.)

**Keywords:** variant PEDV, virus cell adaptation, virus attenuation, genomic analysis

## Abstract

Since 2010, the variant porcine epidemic diarrhea virus (PEDV) has been the etiological agent responsible for the outbreak of porcine epidemic diarrhea (PED) worldwide. In this study, a variant PEDV strain YN1 was isolated, serially propagated on the Vero cells and was characterized for 200 passages. To better elucidate the molecular basis of Vero cell adaptation of variant PEDV strains, we sequenced, compared, and analyzed the full-genome sequences of parental YN1 and passages 15, 30, 60, 90, 144, and 200. The results showed that the variations increased with the viral passage. The nucleotides sequences of non-structural protein (NSP)2, NSP4-7, NSP10, NSP12 and NSP13 genes did not change during the Vero cell adaptation process. After comparison of the variation characteristic of classical, variant virulent/attenuated strains, it was found that attenuation of PEDV virus was associated with 9−26 amino acid (aa) changes in open reading frames (ORF) 1a/b and S protein, early termination in ORF3, 1–3 aa changes in E, M and N protein and some nucleotide sequences’ synonymous mutations. The aa deletion at about 144 aa of S protein could be the attenuation marker for the PEDV. The pig study showed that the early termination in ORF3 was more important for virus cell adaptation than virus attenuation.

## 1. Introduction

Porcine epidemic diarrhea virus (PEDV) belongs to genus *Alphacoronavirus*, family *Coronaviridae*, and it is an enveloped, single-stranded, positive-sense RNA virus with a genome of about 28 kb [1]. The PEDV, a dangerous pathogen for the global swine industry, is the causative agent of porcine epidemic diarrhea (PED), which is characterized by watery diarrhea, dehydration and weight loss in adult pigs, and is lethal in piglets [2].

The first confirmation of PEDV in China was in 1984 [3]. Since 1995, bi-combined killed or attenuated vaccines have been used to combat PED and transmissible gastroenteritis (TGE) in China and its good clinical efficacy has been documented [2,4,5]. However, in October 2010, the outbreak of PED was reported in China, causing huge economic losses [6], and the etiologic agent was identified as the variant PEDV [7]. During the following two and half years, the diseases caused by the variant strains were mainly prevalent in China and led to the death of millions of piglets [8,9]. After first being reported in the United States (US) in May 2013 [10], the Chinese AH2012 strain and other variant strains were also reported in South Korea, Taiwan, Mexico, Germany, and some other regions, causing significant economic losses to the global pig industry [7,8,9,10,11,12,13,14].

The full genome sequence of variant strains is about 28,038 nucleotides (nt) in length. The genome organization is 5′UTR-ORF1a/1b-S-ORF3-E-M-N-3′UTR. Their sizes are about 292 nt; 20,345 nt; 4161 nt; 675 nt; 231 nt; 681 nt; and 1326 nt and 334 nt, respectively [13,14].

The genomes of the classical virulent and corresponding attenuated vaccine strains, such as CV777 and DR13, have been deposited in the GenBank. However, the virulence/attenuation phenotype data coupled with genome changes during the process of Vero cell adaptation of classical and variant PEDV have not yet been reported. Therefore, more studies are required to gain a better understanding of the variant PEDV strains during the Vero cell adaptation process and to unveil the possible factors determining PEDV attenuation. In this study, a variant PEDV strain YN1 was isolated, and it was serially propagated on Vero cells. The virus titer and growth characteristic of strains YN1, YN15, YN60 and YN144 were studied. The full-genome sequence of parental strain YN1 and passages 15, 30, 60, 90, 144, and 200 were sequenced, compared, and analyzed. Additionally, a pig study was implemented to evaluate the virulence of strains YN15 and YN144.

## 2. Materials and Methods

### 2.1. Virus Isolation and Confirmation

Vero cells were purchased from ATCC (Vero, C1008) and cultured in Dulbecco’s modified Eagle’s medium (DMEM, Gibco, Grand Island, NY, USA), supplemented with 10% fetal bovine serum (Invitrogen, Grand Island, NY, USA) at 37 °C with 5% CO_2_. The parental PEDV strain, YN1, was isolated from the intestine of a suckling piglet with acute diarrhea in 2013. The virus was isolated using the previously described methods [15,16]. Briefly, the fresh filtered samples were treated with 10 μg/mL trypsin at 37 °C for 30 min prior to inoculation of Vero cells. DMEM supplemented with 8 μg/mL trypsin was used as maintenance medium. The inoculation protocols were followed according to guidelines [15,16]. Viral propagation was confirmed by daily observation of the cytopathic effect (CPE), reverse transcription (RT)-PCR and indirect immunofluorescence assay (IFA). The infected cells were fixed with cold ethanol as previously described [17] and incubated with our in-house mouse anti-S monoclonal antibody. After washing three times with phosphate-buffered saline (PBS), the infected cells were incubated with 1:100 dilution of FITC-conjugated anti-mouse IgG (Southern Biotech, Birmingham, AL, USA) in the dark.

### 2.2. Serial Passage of PEDV YN1 Strain, Virus Titration, and Virus Growth Characteristic of YN1, YN15, YN60 and YN144

Vero cells were cultured for nearly 24 h to 80% confluence and washed twice with serum-free medium. Then, the cells were inoculated with PEDV strains, YN1, at a multiplicity of infection (MOI) of 0.1 and incubated with serum-free DMEM containing 8 μg/mL trypsin (Invitrogen). The infected cells were lysed by freeze-thawing to harvest the virus at about 36 h post-infection when the CPE was more than 85%. The virus stocks were 10-fold serial diluting and virus titration was performed in 96-well plates in triplicate per dilution. At about 2–3 days post inoculation, the plates were daily observed and the virus titration was determined according to the Reed and Muench method [18] and expressed as plaque-forming unit (PFU)/mL. Vero cells in 12 well plates were inoculated with YN1, YN15, YN60 and YN144 at a MOI of 0.1, and samples were collected at intervals of 6 h to produce one-step growth curves.

### 2.3. The Virulence Experiment of YN15 and YN144

Twelve 10-day-old piglets were used to evaluate the virulence of YN15 and YN144. All the piglets were confirmed to be negative for PEDV, TGEV (transmissible gastroenteritis coronavirus) and RV (rotavirus) using the RT-PCR method reported before [19] and antibody negative for PEDV using PEDV ELISA Kit (Shanghai Shifeng Biotechnology Co. Ltd., Shanghai, China) according to instructions. The piglets were randomly divided into three groups of four. The piglets in different groups were orally administrated with 2 mL YN15 and 2 mL YN144 with the same titer of 10^6^. Median tissue culture infective dose (TCID_50_)/0.1 mL and 2 mL DMEM, respectively. Rectal swabs were collected at day 1 and 5 post inoculation to detect the virus shedding and clinical signs were observed daily. The necropsy was performed at 5 days post inoculation. Tissues from intestinal tract were fixed in formalin and embedded in paraffin wax by standard methods. The immunohistochemical assay was performed by using Anti-Porcine Epidemic Diarrhea Virus (PEDV) spike protein (Hannotech Bioscience, Dongguan, Guangdong, China) as primary antibody. The reaction product was visualized by using a Mouse SABC-AP kit (Boster, Wuhan, Hubei, China) according to instructions [20].

### 2.4. Genome Sequencing

The parental strain YN1 and different passages,* i.e.*, YN15, YN30, YN60, YN90, YN144, and YN200, were diluted with PBS (0.1 M, pH 7.2) for 10% it suspensions. The RNAs were extracted with Trizol reagent (Invitrogen) according to the manufacturer’s instructions. RT was performed by using random primers and TaKaRa RNA PCR Kit (AMV) Ver.3.0 (TaKaRa, Dalian, China) according to the manufacturer’s instructions. The genomes of different passages were sequenced using the next-generation sequencing method as previously reported [21]. The sequences were assembled using the software MEGA v5.05 [22]. The genome sequences of these strains were deposited in the GenBank with the accession numbers KT021227 to KT021233, respectively.

### 2.5. Multiple Alignment and Comparative Analysis

The genome sequences of parental YN1 strain, YN1-derived strains, the virulent DR13 strain (GenBank no. JQ023161.1), and attenuated DR13 strain (GenBank no. JQ023161.1) were aligned by the MEGA v5.05 (K. Tamura, Arizona State University, Phoenix, AZ, USA), and Lasergene v7.2. (DNASTAR Co. Ltd., Madison, WI, USA). The multiple nucleotide and amino acid sequence divergences were generated by Jotun Hein methods by the MegAlign v5.01 (DNASTAR Co. Ltd). The similarity plot was constructed by the program SimPlot, v. 3.5.1 package (S. Ray, Johns Hopkins University, Baltimore, MD, USA) [23] using the two-parameter (Kimura) distance model with a sliding window of 200 bp and step size of 20 bp.

### 2.6. Statistical Analysis

Statistical analysis was performed using Origin Pro 8 (Northampton, MA, USA) and SPSS statistics 17.0 (Chicago, IL, USA) software. Differences between medians in each of the two groups were determined using a paired student *t* test. Two-sided probability values <0.01 were considered to indicate statistical significance.

## 3. Results

### 3.1. Virus Isolation, Identification and Virus Growth Characteristic of YN1, YN15, YN60 and YN144

**Figure 1:**
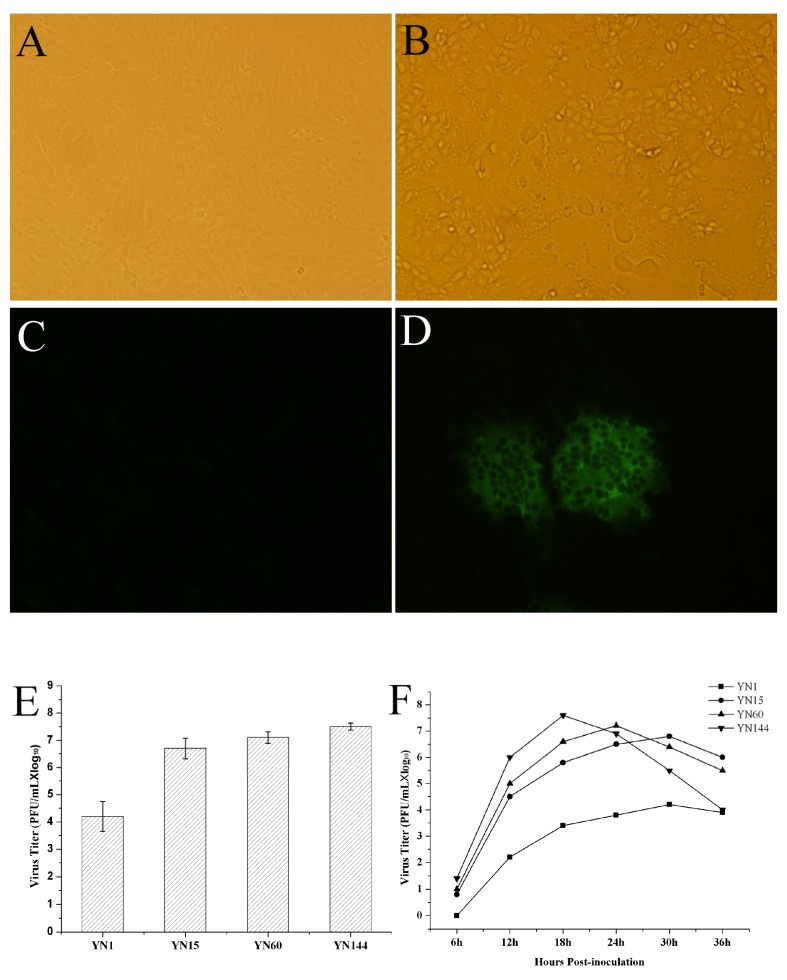
Virus identification, virus titration and propagation kinetic. (**A**) Control Vero cells; (**B**) The cytopathic effects (CPE) of Vero cells infected with YN15 at 24 h post-inoculation; (**C**) Blank control cells in immunofluorescence assay (IFA); (**D**) Fluorescent signal in porcine epidemic diarrhea virus (PEDV)-infected cells; (**E**) Virus titer of YN1, YN15, YN60 and YN144; (**F**) One-step growth curve of YN1, YN15, YN60 and YN144 in Vero cells.

The PEDV strainYN1 was successfully isolated. The strain was subsequently confirmed by RT-PCR and IFA. The propagation kinetics of different passages of PEDV in Vero cells were constructed by calculation of PFU per mL. As shown in Figure 1B, obvious CPE was observed at 36 h post-inoculation (hpi). Significant fluorescence signals could be identified at 30 hpi (Figure 1D). The viral titers were about 4.2 log_10_, 6.8 log_10_, 7.2 log_10_, and 7.6 log_10_ PFU/mL for YN1, YN15, YN60 and YN144 (Figure 1E), respectively. The one-step growth curves showed that the viral titers of YN1 and YN15 were 2.2 log_10_, 3.4 log_10_, 3.8 log_10_, 4.2 log_10_, 3.9 log_10_ PFU/mL and 4.5 log_10_, 5.8 log_10_, 6.5 log_10_, 6.8 log_10_, 6 log_10_ PFU/mL at 12, 18, 24, 30, and 36 hpi, respectively. The viral titers of YN60 and YN144 were 5.0 log_10_, 6.6 log_10_, 7.2 log_10_, 6.4 log_10_, 5.5 log_10_ PFU/mL and 6.0 log_10_, 7.6 log_10_, 6.9 log_10_, 5.5 log_10_, 4.0 log_10_ PFU/mL at 12, 18, 24, 30, and 36 hpi, respectively. The viral titer of YN1, YN15, YN60, and YN144 peaked at 4.2 log_10_ PFU/mL at 30 hpi, 6.8 log_10_ PFU/mL at 30 hpi, 7.2 log_10_ PFU/mL at 24 hpi, and 7.6 log_10_ PFU/mL at 18 hpi, respectively (Figure 1F). We concluded that YN15, YN60 and YN144 were cell culture adapted compared to the original YN1 strain because their total and peak virus titers were higher and growth curves shorter than the YN1 strain (Figure 1E,F). These results suggested that the nucleotide and amino acid (aa) changes of passages YN15 and later might contribute to the cell culture adaptation of these strains.

### 3.2. Pathogenicity Analysis of YN15 and YN144

**Figure 2:**
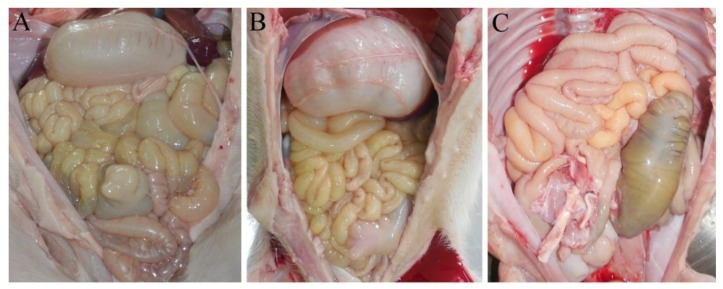
The necropsies results of pigs infected with YN15 (**A**), YN144 (**B**) and the control group (**C**).

**Figure 3:**
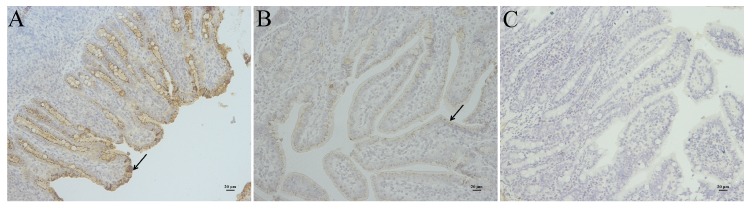
The immunohistochemical (IHC) assay of pigs infected with YN15 (**A**) and YN144 (**B**) as well as control group (**C**) are indicated. Arrows indicate positive signal of IHC assay in the intestinal tissue. Original magnification ×200.

**Figure 4:**
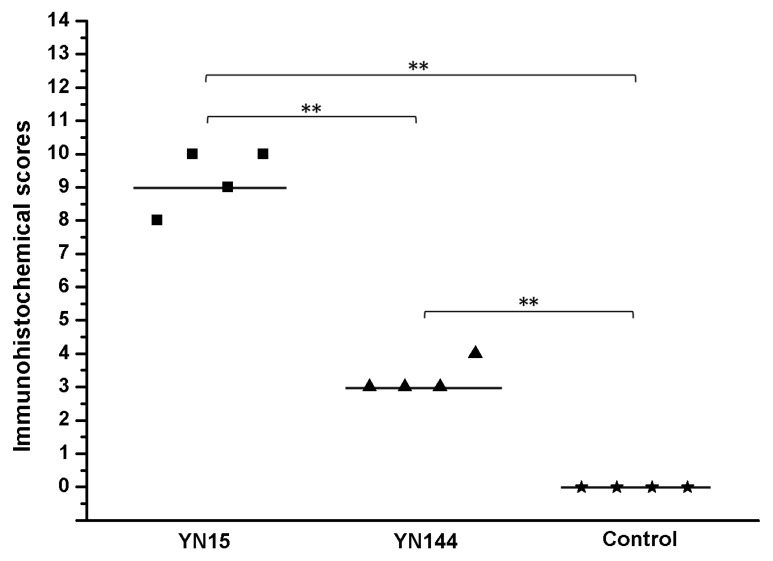
The immunohistochemical (IHC) results were scored by the distribution or extent of positive signal of IHC assay, each data point represents an individual animal in each treatment group, and median values are indicated by horizontal bars. ** *p *< 0.01.

All the four piglets infected with YN15 strain showed watery diarrhea and significantly emaciated body. The piglets infected with YN144 showed no watery diarrhea and their health was as good as those of piglets in the control group. The PEDV was detected in rectal swabs of YN15 group at day 1 and 5 post inoculation by RT-PCR, while undetected in rectal swabs of YN144 and control groups (data not shown). The necropsies and immunohistochemical (IHC) results were observed to identify the virulence difference and infection characteristics of YN15 and YN144. The necropsies of YN15- and YN144-infected as well as control groups of pigs were demonstrated in Figure 2A–C, respectively. The intestines of four pigs infected with YN15 showed typically fluidic, distended, and yellow water-like content (Figure 2A); however, pigs in the YN144 group were as normal as those in the control group (Figure 2B,C). According to the IHC score system [24], the IHC score of tissue of YN15-infected pig (Figure 3A), YN144- infected pig (Figure 3B) and pig in control group (Figure 3C) was 9.25 ± 0.75, 3.25 ± 0.375 and 0, respectively. The IHC scores in the YN15 treated group were significantly different compared to the YN144 treated group (*p* < 0.01; Figure 4). The clinical symptoms, necropsies and IHC assay results of the pig infection experiment showed that the YN15 is a virulent PEDV strain and YN144 is an attenuated PEDV strain. The comparison of genomic sequences of passages YN1 and later could lay the foundation for better understanding the molecular mechanic of PEDV attenuation.

### 3.3. Amino Acid Changes during Passage

The full genome nucleotide alignment of different passages revealed that the number of nucleotides’ variation increased with the passage, in which there were some synonymous and non-synonymous mutations. The most synonymous mutations happened when comparing the YN1 with YN15. Comparison of YN1 with different passages of Vero cell-adapted viruses (YN15, YN30, YN60, YN90, YN144, and YN200), the number of aa changes were 15, 20, 22, 25, 33, and 39, respectively (Table 1). In 33 aa changes in the YN144, there were only 6 aa changes (21.2%, 7/33) in non-structural protein open reading frames (ORF)1a/b, which accounted for 72.6% of the genome, whereas 81.8% aa change occurred in structural proteins, which only occupied 27.4% of the genome. In structural proteins, S protein had the highest change rate, about 36.4% (12/33). It is worth noting that there were 3 aa deletions (at 144 aa, 145 aa, and 1197 aa) in S protein. In ORF3 gene, there was a nucleotide deletion, resulting in early termination of ORF3 at 145 aa, and before termination, there were 8 aa changes from 138 aa to 145 aa. In the other three proteins,* i.e.*, E, M and N, there were 2 aa changes (Table 1).

**Table 1:** The amino acid (aa) variation of non-structural and structural proteins during the serial passage (The mutated amino acids are underlined).

Gene	aa position	1	15	30	60	90	144	200
ORF1a/b	509	P	P	P	P	P	S	S
	1566	I	I	T	T	T	T	T
	2436	L	L	L	L	L	L	F
	2925	H	H	H	H	H	Y	Y
	3505	V	V	V	V	V	E	E
	4832	V	V	V	V	F	F	F
	5951	A	A	A	A	A	A	P
	6136	L	F	F	F	F	F	F
	6137	V	S	S	S	S	S	S
S	144	T	T	-	-	-	-	-
	145	G	G	-	-	-	-	-
	372	Y	Y	Y	Y	Y	Y	H
	405	D	D	D	D	G	G	G
	428	G	G	G	A	A	A	A
	490	R	T	T	T	T	T	T
	722	S	S	S	S	S	R	R
	779	T	T	T	T	N	N	N
	825	Q	Q	H	H	H	H	H
	887	S	S	S	S	S	S	R
	968	S	A	A	A	A	A	A
	1045	H	Q	Q	Q	Q	Q	Q
	1165	D	N	N	N	N	N	N
	1197	H	-	-	-	-	-	-
	1210	F	Y	Y	Y	Y	Y	Y
	1218	S	G	G	G	G	G	G
	1304	I	I	I	I	I	L	L
	1354	C	C	C	C	C	F	F
ORF3	138–141	YYDG	FMTA	FMTA	FMTA	FMTA	FMTA	FMTA
	142–145	KSIV	NPL *	NPL *	NPL *	NPL *	NPL *	NPL *
E	53	I	I	I	I	V	V	V
	70	P	P	P	S	S	S	S
M	187	V	V	V	V	V	F	F
	214	A	S	S	S	S	S	S
	226	V	V	V	V	V	V	F
N	112	V	V	V	V	V	F	F
	379	V	V	V	V	V	A	A
	391	S	S	R	R	R	S	S

*: termination codon.

### 3.4. Sequence Homology and Variation Characteristic of strainsYN1, YN15, YN30, YN60, YN120, YN144 and YN200

The genome sequence similarity between the parental virus, YN1, and its virulent, attenuated counterparts were between 99.6% to 99.8% at the nucleotide level, and 99.7% to 99.9% at the aa level. The genome sequence homologies between cell-adapted virulent virus YN15 and its attenuated counterparts were 99.9% at nucleotide and aa level. For Vero cell-adapted YN15, the nucleotide sequences were first mutated in the ORFs (*NSP11*, *S*, *ORF3*, and *M* gene). The more important observation is that, from passage 15, all Vero cell-adapted passages were consistently terminated early in ORF3 and had one aa deletion at 1197 aa of S protein. The nucleotide sequences of ORFs progressively mutated slowly. From YN30, there were two aa deletions at 144 aa and 145 aa of S protein. The aa change numbers in NSP1, NSP3, NSP9, NSP11, S, ORF3, E, M, and N in YN144 and YN200 were 1, 1, 1, 2, 12, 8, 2, 2, 2 and 2, 1, 1, 3, 15, 8, 2, 3, 2, respectively (Table 2).

**Table 2:** Amino acid changes numbers and change rates between parental virulent YN1 and different Vero cell-adapted passages.

ORFs	Encoded Proteins	1/15	1/30	1/60	1/90	1/144	1/200
ORF1a/b	NSP1	0(0) *	1(0.06)	1(0.06)	1(0.06)	1(0.06)	2(0.12)
	NSP3	0(0)	0(0)	0(0)	0(0)	1(0.36)	1(0.36)
	NSP9	0(0)	0(0)	0(0)	1(0.11)	1(0.11)	1(0.11)
	NSP11	2(0.39)	2(0.39)	2(0.39)	2(0.39)	2(0.39)	3(0.58)
S	S protein	6(0.43)	7(0.50)	8(0.58)	11(0.79)	12(0.87)	15(1.08)
ORF3	ORF3	8(3.55)	8(3.55)	8(3.55)	8(3.55)	8(3.55)	8(3.55)
E	E protein	0(0)	0(0)	1(1.30)	2(2.60)	2(2.60)	2(2.60)
M	M protein	1(0.44)	1(0.44)	1(0.44)	1(0.44)	2(0.72)	3(1.08)
N	N protein	0(0)	1(0.23)	1(0.23)	1(0.23)	2(0.45)	2(0.45)

* The amino acid change numbers and change rates in the corresponding proteins (%).

### 3.5. The Conserved and Highly Mutable Proteins

**Figure 5:**
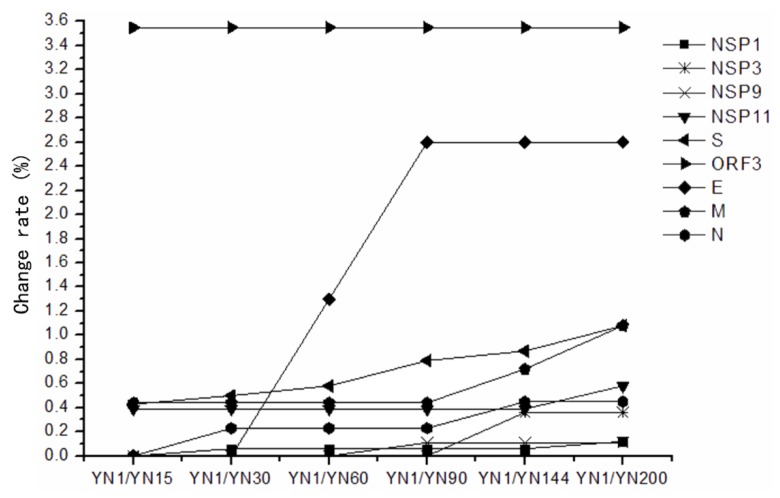
The change rate of non-structural and structural proteins of different passages compared with their virulent parental strain YN1. The change rate is the percentage of changed amino acid in their corresponding proteins (%). Non-structural proteins: NSP1, NSP3, NSP9, and NSP11 proteins; Structural proteins: S, ORF3, E, M, and N proteins.

In the non-structural genes, *NSP2*, *NSP4-7*, *NSP10*, *NSP12*, *NSP13* had no nucleotide or amino acid change, thereby suggesting that these proteins were unrelated to Vero cell-adaptation of variant PEDV. The other non-structural genes, *NSP1*, *NSP3*, *NSP9*, *NSP11* were less conserved and aa change rates of these proteins in YN200 were 0.12%, 0.36%, 0.11%, and 0.58%, respectively. In the structural genes, *ORF3* was the most mutable gene. Besides early termination at 145 aa, ORF3 also had 8 aa changes just before the terminated position. The E protein was a highly changeable protein, and from passage 90, it became stable. S and M proteins were also highly changeable proteins. The S protein was increasingly changed, while the M protein reached a plateau from passage 15 to 90. Then, from passage 90, the M protein changed quicker than the S protein, and they reached the same change rate at passage 200 (1.08%). The N protein was a conserved protein, with only two aa changes, and the change rate was 0.45% (Table 2) (Figure 5).

### 3.6. The Highly Variable Regions

**Figure 6:**
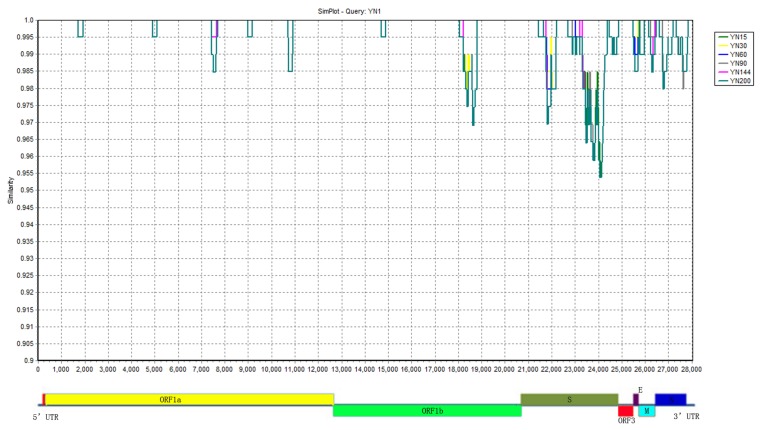
Similarity plot of the full-genome of all Vero cell-adapted passages with parental YN1. The similarity plot was constructed using the two-parameter (Kimura) distance model with a sliding window of 200 bp and step size of 20 bp. The vertical and horizontal axes indicate the percent nucleotide similarity and nucleotide position (bp) in the graph, respectively.

During the serial passages on the Vero cells, the genome mutations mainly occurred in the structural proteins regions. Based on the similarity plot of different passage viruses with their parental strain YN1, several highly variable regions were identified. They were located at 5951–6137 aa in NSP11, 144–490 aa and 722–1218 aa in S, 138–145 aa in ORF3, 53–70 aa in E, 187–226 aa in M, and 379–391 aa in N (Figure 6).

### 3.7. Comparison of Variation Characteristic of the Classical and Variant Virulent/Attenuated Strains

**Figure 7:**
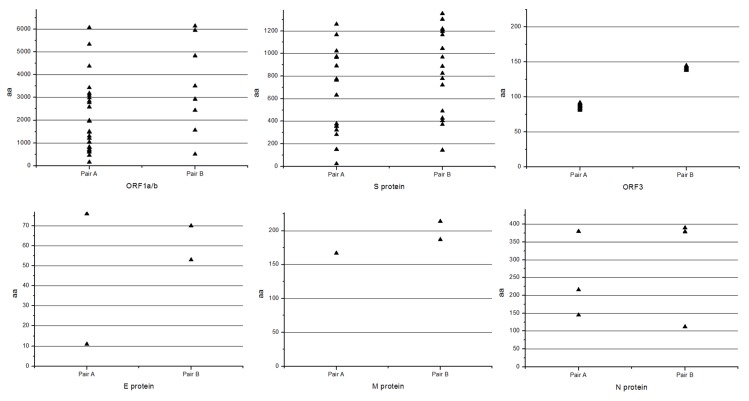
The amino acid change in ORF1a/b, S, ORF3, E, M, and N proteins. **Pair A**: Virulent DR13/Vaccine DR13; **Pair B**: YN1/YN144.The X-axis represents the aa changes in the vaccine DR13 and YN144, and the Y-axis represents aa change position. The upper triangle represents one mutated amino acid.

The virulent DR13 and attenuated DR13 were clustered in the same group with classical PEDV strains, while the YN1 and YN144 were clustered into variant PEDV strains. Table 3 illustrates that the variation characteristic of attenuation of classical and variant PEDV strains is 9–26 aa changes in ORF1a/b and S protein, 1–3 aa deletions in S protein, early termination and 8–12 aa changes in ORF3, 1–3 aa changes in E, M and N protein (Table 3), and some nucleotide sequences’ synonymous mutations. To better understand the aa change positions of the ORFs, please see Figure 7. The figure presents the concrete aa change positions in these proteins and shows that the aa change positions in the two pairs of PEDV strains (Virulent DR13/Vaccine DR13 and YN1/YN144) are not same, whereas the aa change numbers in these proteins are similar (Figure 7). Some aa changes in ORF1a/b, S, and ORF3, and a few aa changes in E, M, and N proteins were identified. Specifically, they all had an aa deletion at about 144 aa of S protein and early termination in ORF3, and an aa deletion at about 144 aa of S protein could be the attenuation marker for the PEDV.

**Table 3:** Comparison of the variation in genome of classical and variant virulent/attenuated strains.

Genes	Number of Amino Acid Change and Deletion when Compared Virulent DR13 with Attenuated DR13	Number of Amino Acid Change and Deletion when Compared YN1 with YN144
ORF1a/b	26	9
S	18; One aa deletion at 151aa	16; Three deletions at 144–145aa, and 1197aa
ORF3	12; Early termination at 92aa	8; Early termination at 144aa
E	2	2
M	1	2
N	3	2

## 4. Discussion

Currently, PED caused by a variant strain is a severe problem for the global pig industry [9,10,11,12,13,14]. It was predominant in China and led to millions of piglets’ deaths [6,7]. Although the classical bi-combined killed or attenuated vaccines had good clinical efficacy against traditional strain-related PED [15,25], they could not provide enough protection against the new PED epidemic [7]. Thus, unveiling the genomic characterization of the variant PEDV was very important for discovering the pathogenesis, and Vero cell-adaptation, attenuation and vaccine development.

The classical strains’ derived vaccine had good efficacy against PED caused by the traditional PEDV strains. The differences in virulence and genome sequences between the parental and their derived attenuated strains were extensively studied [15,25,26]. These studies helped us better understand the variation characteristic of the Vero cell-adaptation process of PEDV strains. In this study, the passages YN15 and later were cell-culture-adapted compared to the original YN1 strain, and the viral titers of YN1, YN15, YN60, and YN144 were continuously increasing. These results suggested that the nucleotide and aa changes of passages YN15 and later might contribute to the cell culture adaptation of these strains. The comparison of genomic sequences of passages of YN1 and later could shed some light on the molecular mechanic of PEDV adaptation. In the virulence evaluation experiment of YN15 and YN144, the YN15 strain which was cell-adapted and was terminated early in the ORF3 gene could cause watery diarrhea in pigs and could colonize in the intestines (Figure 2A and Figure 3A), while no clinical signs in YN144-infected pigs were observed, indicating that infection efficacy was enormously decreased (Figure 2B and Figure 3B). The clinical symptoms, necropsies and immunohistochemical assay results of the pig infection experiment showed that the YN15 is a virulent PEDV strain and YN144 is an attenuated PEDV strain. The comparison of genomic sequences of passages of YN1 and later could lay the foundation for better understanding the molecular mechanic of PEDV attenuation. In the other clinical trials, the YN144 was safe for pregnant sows and newborn piglets, and could provide enough protection to sows and their piglets against a new PED. The genome sequence characteristic of variant PEDV in the USA during the* in vitro* Vero-cell adaptation process was reported [27]. The two isolates (ISU13-19338E and ISU13-22038) were serially propagated in cell culture for over 30 passages and characterized for the first 10 passages. Nucleotide and aa changes of two PEDV isolates, ISU13-19338E and ISU13-20338, after 9 passages in cell culture were different. When the nucleotide and aa changes of YN1 were compared after 14 passages in cell culture with two isolates, ISU13-19338E and ISU13-20338, and after 9 passages in cell culture, none of the nucleotide or aa changes were observed to be the same. However, nucleotide and aa changes of YN1, ISU13-19338E and ISU13-20338 during the first few passages in cell culture were first observed in *OR1ab* and *S* gene. The virulence/attenuation phenotype data of different passages of these two isolates (ISU13-19338E and ISU13-22038) were not presented in their study. So, further research is needed to study the virulence/attenuation phenotype characteristic of variant PEDV. In our study, the isolates were serially propagated in cell culture for over 200 passages and characterized for the 200 passages. The attenuation of YN144 was evaluated in the pig model. The genome sequences comparison between the variant parental strains with their Vero cell-adapted passages could therefore help us to better understand the re-emerging variant PEDV. Moreover, the genome sequences comparison between classical and variant virulent/attenuated strains unveiled some common variation characteristics between these two pairs of PEDV strains.

Generally, virus passage* in vitro *in different species would result in attenuation during the process to adapt to new cell lines. So, understanding Vero cell adaptation of variant PEDV might help us to develop a new vaccine against the re-emerging PED. During the cell adaptation process, some mutations are responsible for cell adaptation, some for attenuation, and some for both [28]. Normally, the mutations occurring in the early stage of passages are related to adaptation. In this study, the cell cultures of parental strain YN1 and different passages,* i.e.*, YN15, YN30, YN60, YN90, YN144, and YN200 were collected for sequencing. The sequencing results would be more specific if we used the plaque purified isolates as the sequencing samples. In the early passage—YN15—only the nucleotide sequences change of *NSP11*, *S*, *ORF3*, and *M* were noted. S glycoprotein (surface antigen) is the major surface protein in PEDV, which is responsible for viral receptor binding [29,30,31]. M protein was an important viral assembly process-associated protein [32]. *ORF3* was the only accessory gene of PEDV and the variation of *ORF3* during the cell culture adaptation process resulted in the virulence decrease of the PEDV [33], as happened in TGEV [34]. It is believed that the variation of *ORF3* between parental virulent strains and attenuated strains could be markers for both attenuation and cell adaptation of the virus [26,29,35]. Thus, the aa changes in S protein and M protein and early termination in ORF3 could be related to cell adaptation of PEDV. 

In the non-structural genes, such as *NSP2*, *NSP4-7*, *NSP10*, *NSP12*, *NSP13*, no nucleotide or amino acid change were noted, suggesting that these proteins are unrelated to variant PEDV Vero-cells adaptation. The nucleotides of other non-structural genes, *NSP1*, *NSP3*, *NSP9*, *NSP11*, were increasing changing during the passage. The nucleotides changes of these genes might play an important role in the transcriptional regulation and virus adaption and virulence. The aa change rates in the structural proteins were higher than those of the non-structural proteins, and the *ORF3* was the most mutable gene. Besides early termination at 145 aa in ORF3 protein, it also had 8 aa changes just before the terminated position. The aa changes before the early termination position of ORF3 seemed to be a reason for virus adaptation. The *ORF3* was therefore believed to be the most important gene responsible for the Vero cell adaptation of PEDV through* in vitro *passage. The E, S and M protein were highly changeable proteins, but after the 90th passage, the E protein became stable, the S protein was increasingly changed and the M protein reached a plateau from passage 15–90. Then from passage 90, the M protein changed faster than the S protein, and they reached the same mutation rate level at passage 200 (1.08%). The N protein was a conserved protein, with only two aa changes, and the mutation rate was 0.45% (Table 2) (Figure 1).

Table 3 shows that the variation of attenuation in both classical and variant PEDV strains can be characterized by 9–26 aa changes in ORF1a/b and S proteins, 1–3 aa deletions in S protein, early termination and 8–12 aa changes in ORF3, 1–3 aa changes in E, M and N proteins, and the nucleotide sequences’ synonymous mutations of genome (RNA) sequence might also be involved in attenuation. However, to explore which nucleotides mutations contributed to the attenuation of PEDV we could use the reverse genetic approach. The early termination of ORF3 could be the attenuation and cell adaptation marker of the PEDV [26,29,35], whereas in our pig infection experiment, the ORF3 early termination strain, YN15, was still virulent to piglets. Thus, we deduced that the early termination in ORF3 was more important for virus cell culture adaptation than virus attenuation, and aa deletion at about 144 aa of S protein could be the attenuation marker for the PEDV. 

The* in vivo* challenge experiment implied the attenuation of YN144. No diarrhea was observed and no virus shedding was detected in YN144 infected piglets. Based on a previous pig study [36], fecal virus shedding could be detected but no microscopic lesions or IHC staining could be observed in early stage of infection. In this study, the virus was undetected in rectal swabs of YN144 group although IHC staining was observed in YN144 group. This is because viruses are colonized in intestinal epithelial cells. If the intestinal epithelial cells are not destroyed, the viruses cannot be released from intestinal epithelial cells into feces and they cannot be detected by RT-PCR. The YN144 is an attenuated PEDV strain which cannot cause the damage of the intestinal epithelial cells, so YN144 could not be detected from fecal samples, although their presence can be shown in the IHC results.

As a live attenuated vaccine candidate, the potential genetic stability of viruses is our main concern. Through the genome sequence comparison between parental virulence strains and their different passages, the aa changes were increasing during the serial passage, whereas from passage 60, the strains (YN90, YN144, and YN200) consistently have aa deletion in the S protein and early termination in ORF3, thereby indicating the genetic stability of YN144 which is needed for further* in vivo *evaluations.

## 5. Conclusions

In conclusion, to unveil the variation characteristic of variant PEDV strains during the Vero cell-adaptation process, we compared the full genome sequences of the variant virulent parental/attenuated strains. To better understand the attenuation characteristic of PEDV, we compared the full genome sequences of classical and variant parental virulent/attenuated strains. The results showed that in the variant strains of PEDV, the variations increased with the passage. The NSP2, NSP4-7, NSP10, NSP12, NSP13 were not related to the Vero cell-adaptation process of variant PEDV strains. The PEDV attenuation was associated with 9–26 aa changes in ORF1a/b and S, early termination in ORF3, 1–3 aa changes in E, M and N protein and some nucleotide sequences’ synonymous mutations. The aa deletion at about the 144 aa position of S protein could be the attenuation marker for the PEDV. The pathogenicity experiment in the pig model showed that early termination in ORF3 was more important for virus adaptation than virus attenuation. These findings could help us better understand the mechanism of cell culture adaptation, attenuation of variant PEDV, and thus provide clues for further vaccine development.
